# Supplementary material for: Bacteriophage-antibiotic combination therapy against extensively drug-resistant Pseudomonas aeruginosa infection to allow liver transplantation in a toddler
Source: Nat Commun. 2022 Sep 29;13:5725. doi: 10.1038/s41467-022-33294-w (PMC9523064; doi:10.1038/s41467-022-33294-w)
Supplement: Supplementary file 1 — Supplementary Information [file 41467_2022_33294_MOESM1_ESM.pdf]

Bacteriophage-antibiotic combination therapy against extensively drug-resistant *Pseudomonas aeruginosa* infection to allow liver transplantation in a toddler

Supplementary Information

Description of Supplementary Information

Supplementary Figures – see below

- Supplementary Figure 1 - Intraoperative intra-abdominal phage therapy
- Supplementary Figure 2 - Antibiotic susceptibility phenotype (a) and related genes (b) of seven *Pseudomonas aeruginosa* isolates retrieved before ($Pa1_{BS}$) and during ($Pa2_{BR}$-$Pa7_{BS}$) phage therapy
- Supplementary Figure 3 - Growth kinetics of the 7 *P. aeruginosa* isolates
- Supplementary Figure 4 - *Galleria mellonella* assay

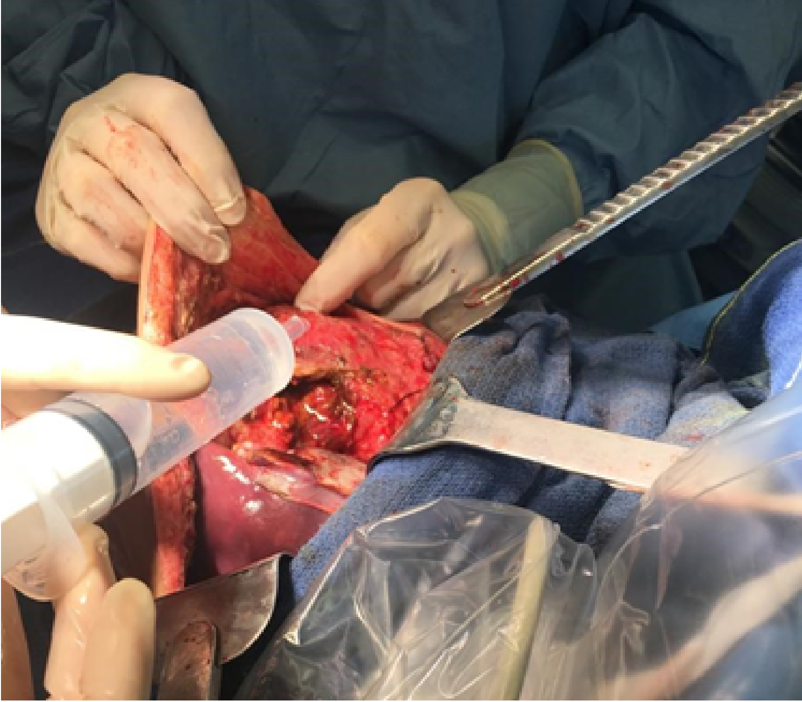

Supplementary Figure 1 | Intraoperative intra-abdominal phage therapy. Instillation of 250 mL of BFC1, containing 10^7 plaque forming units (pfu)/mL of phages ISP, PNM and 14-1, for 30 minutes, during the anhepatic phase of deceased-donor liver transplantation.

a

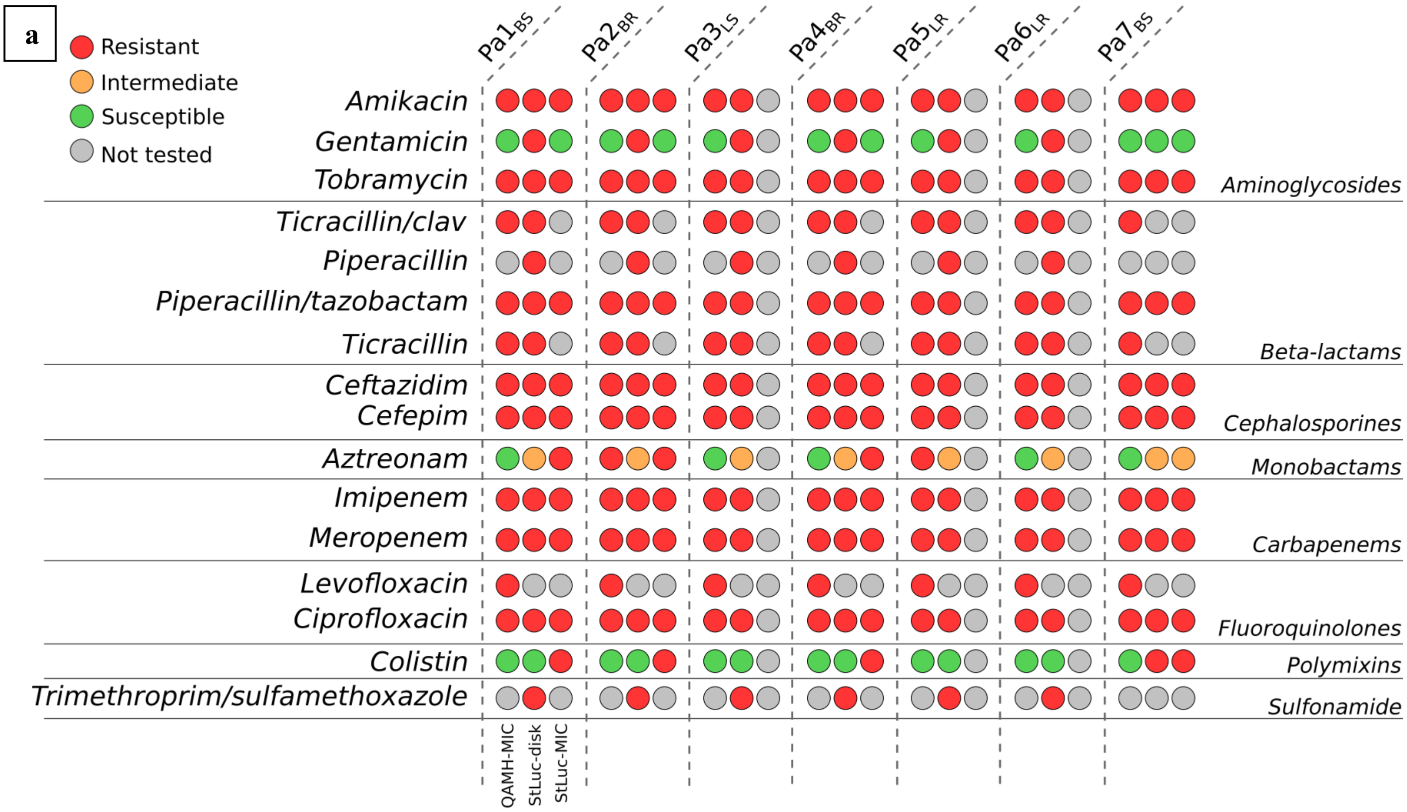

QAMH-MIC, minimum inhibitory concentrations determined at the Queen Astrid Military Hospital ; StLuc-MIC, minimum inhibitory concentrations determined at Saint-Luc University Hospital ; StLuc-disk, antibiotic susceptibility determined at Saint-Luc University Hospital by disk diffusion method

b

Isolate	Number of AMR genes	Aminoglycoside : **aac(6')-29a**	Aminoglycoside : **aac(6')-29b**	Kanamycin : **aph(3')-IIb**	Beta-lactam : **blaOXA-395**	Cephalosporin : **blaPDC-55**	Carbapenem : **blaVIM-2**	Chloramphenicol : **catB7**	Fluoroquinolone : **crpP**	Fosfomycin : **fosA-354827590**	Sulfonamide : **sul1**
**Pa1$_{BS}$**	6	-	-	+	+	+	-	+	+	+	-
**Pa2$_{BR}$**	9	-	+	+	+	+	+	+	+	+	+
**Pa3$_{LS}$**	10	+	+	+	+	+	+	+	+	+	+
**Pa4$_{BR}$**	10	+	+	+	+	+	+	+	+	+	+
**Pa5$_{LR}$**	6	-	-	+	+	+	-	+	+	+	-
**Pa6$_{LR}$**	8	+	-	+	+	+	+	+	+	+	-
**Pa7$_{BS}$**	8	+	-	+	+	+	+	+	+	+	-

AMR, antimicrobial resistance

Supplementary Figure 2 | Antibiotic susceptibility phenotype (a) and related genes (b) of seven *Pseudomonas aeruginosa* isolates retrieved before (Pa1$_{BS}$) and during (Pa2$_{BR}$-Pa7$_{BS}$) phage therapy. The nomenclature of these isolates consists of : [Pa] for *P. aeruginosa*; [number] for chronological order of isolation; [first letter: B/L] for origin of isolate, either from Blood culture or Liver abscess pus culture; [second letter: S/R] for phenotypic Susceptibility or Resistance of the isolate to phage PNM. *P. aeruginosa* isolates Pa1$_{BS}$, Pa2$_{BR}$, Pa4$_{BR}$, and Pa7$_{BS}$ were retrieved from blood samples harvested on days 57, 58, 61, and 65 post-LDLT, respectively. *P. aeruginosa* isolates Pa3$_{LS}$, Pa5$_{LR}$, and Pa6$_{LR}$ were isolated from liver abscess pus on days 58 (Pa3$_{LS}$) and 61 (Pa5$_{LR}$ and Pa6$_{LR}$). Minimum Inhibitory Concentrations were determined at the Queen Astrid Military Hospital (QAMH-MIC) using VITEK 2 technology and the ADVANCED EXPERT SYSTEM (both bioMérieux Benelux, Brussels, Belgium), and were determined at Saint-Luc University Hospital (StLuc-MIC) through automated microdilutions (Phoenix, Becton Dickinson, Franklin Lakes, NJ, USA). Antibiotic susceptibility was also assessed through disk diffusion method in Saint-Luc University Hospital (StLuc-disk) using Adagio reading technology (Bio-Rad, Marnes-la-Coquette, France).

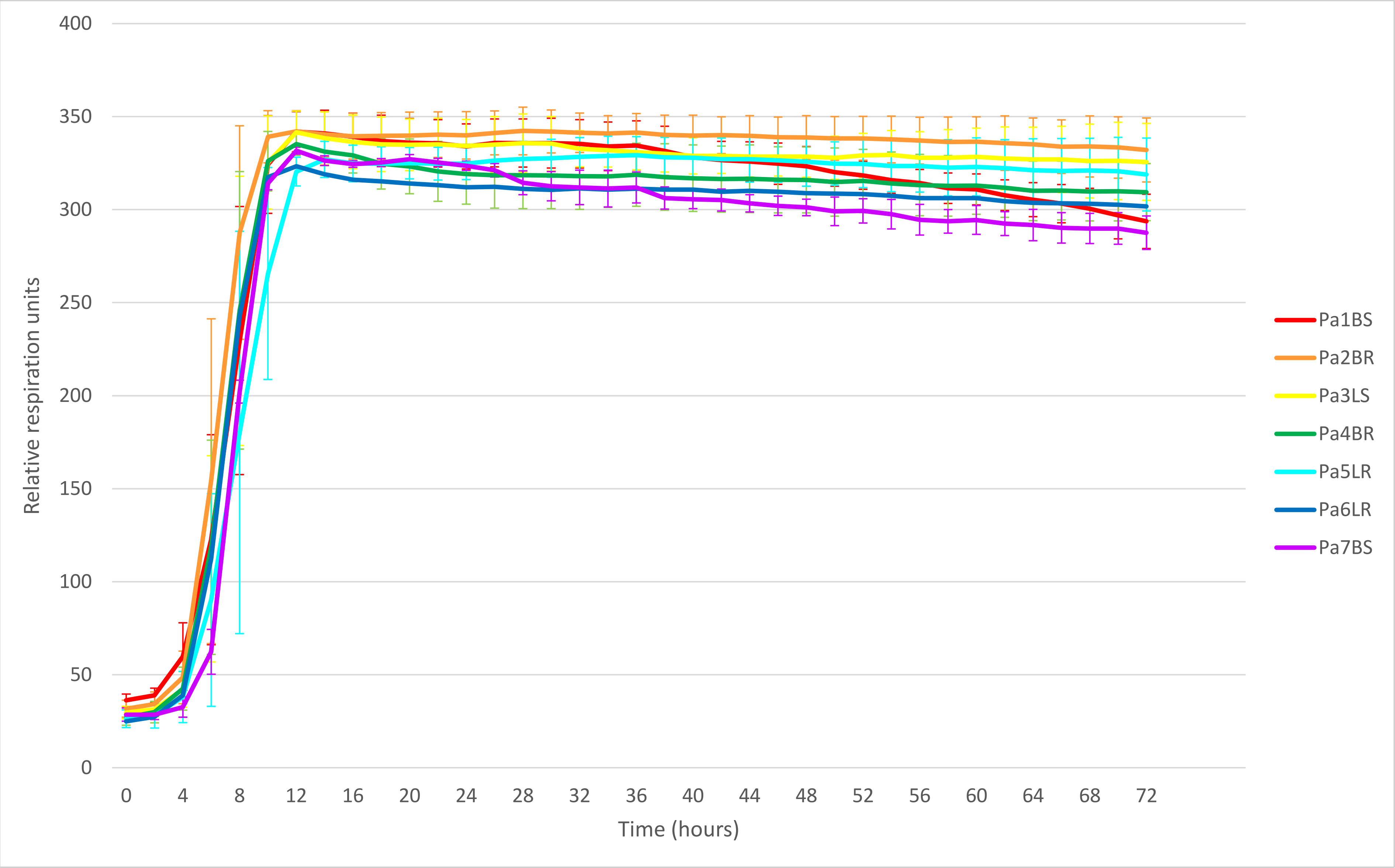

Supplementary Figure 3 | Growth kinetics of the 7 *P. aeruginosa* isolates. Growth kinetics were measured using an OmniLog® system. Bacterial proliferation is presented through relative units of cellular respiration. Results are presented as mean values of three experiments (biological replicates) with error bars representing the standard deviations of the means.

a

Category	Description	Score
Activity	No activity despite stimulation	0
	Minimal activity upon stimulation	1
	Normal activity upon stimulation	2
	Spontaneously active without stimulation	3
Melanization	Complete melanization : black larvae	0
	Brown larvae with darker spots	1
	≥3 spots on beige larvae	2
	<3 spots on beige larvae	3
	No melanization : cream white larvae	4
Survival	Dead	0
	Alive	2
Total	Sum of all categories, 0 to 9	

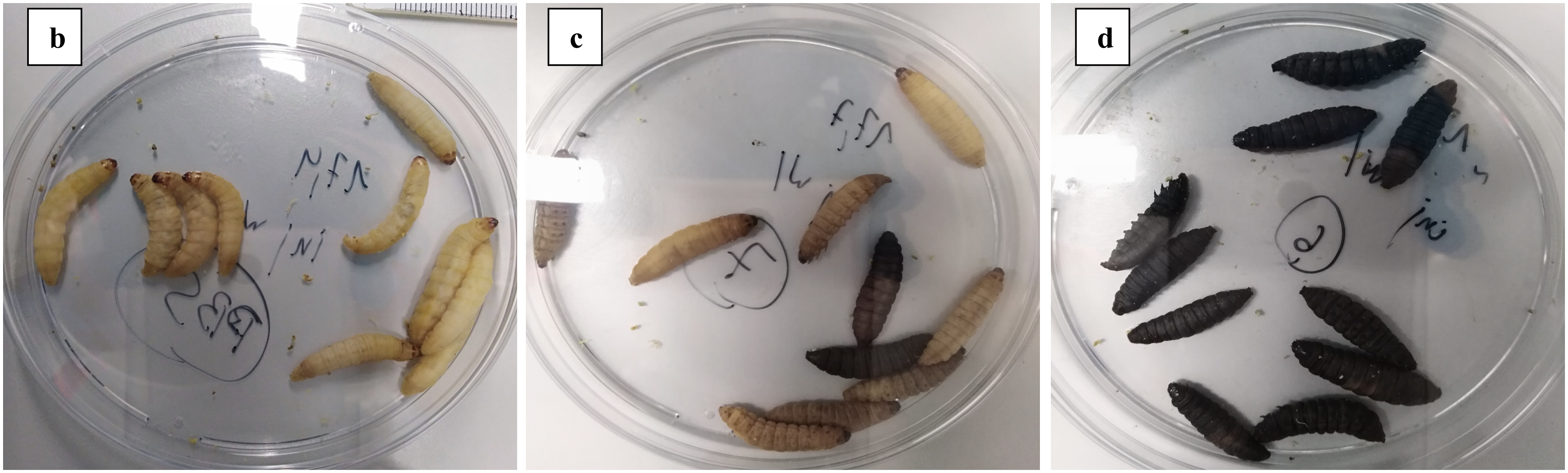

Supplementary Figure 4 | *Galleria mellonella* assay a An activity score was used to assess the *Galleria mellonella* (Gm) larvae health status after inoculation with each *Pseudomonas aeruginosa* isolate by assigning scores according to three major observation categories: larvae activity, melanization and survival. **b** Healthy larvae are cream-colored, have noticeable tonus and are spontaneously mobile. **c** as infection progresses, mobility weakens and color darkens to varying shades of beige and brown, with noticeable inter-individual variability highlighting the need to use batches of several larvae. **d** at some point in time, all larvae die then reach a total activity score of 0 upon complete melanization (black color).
