# Supplementary material for: Bacteriophage-antibiotic combination therapy against extensively drug-resistant Pseudomonas aeruginosa infection to allow liver transplantation in a toddler
Source: Nat Commun. 2022 Sep 29;13:5725. doi: 10.1038/s41467-022-33294-w (PMC9523064; doi:10.1038/s41467-022-33294-w)
Supplement: Supplementary file 3 — Description to Additional Supplementary Information [file 41467_2022_33294_MOESM3_ESM.pdf]

Description of Additional Supplementary Files

Supplementary Data 1 - Single Nucleotide Polymorphisms (SNP) present in the patient's P. aeruginosa isolates.

Supplementary Data 2 - Structural Variations (SV) present in the patient's P. aeruginosa isolates.
