# Supplementary material for: Bacteriophage-antibiotic combination therapy against extensively drug-resistant Pseudomonas aeruginosa infection to allow liver transplantation in a toddler
Source: Nat Commun. 2022 Sep 29;13:5725. doi: 10.1038/s41467-022-33294-w (PMC9523064; doi:10.1038/s41467-022-33294-w)
Supplement: Supplementary file 6 — Reporting Summary [file 41467_2022_33294_MOESM6_ESM.pdf]

nature portfolio

Corresponding author(s): Brieuc Van Nieuwenhuyse

Last updated by author(s): Sep 9, 2022

Reporting Summary

Nature Portfolio wishes to improve the reproducibility of the work that we publish. This form provides structure for consistency and transparency in reporting. For further information on Nature Portfolio policies, see our Editorial Policies and the Editorial Policy Checklist.

Statistics

For all statistical analyses, confirm that the following items are present in the figure legend, table legend, main text, or Methods section.

n/a	Confirmed	
☐	☒	The exact sample size (*n*) for each experimental group/condition, given as a discrete number and unit of measurement
☐	☒	A statement on whether measurements were taken from distinct samples or whether the same sample was measured repeatedly
☐	☒	The statistical test(s) used AND whether they are one- or two-sided<br>*Only common tests should be described solely by name; describe more complex techniques in the Methods section.*
☒	☐	A description of all covariates tested
☒	☐	A description of any assumptions or corrections, such as tests of normality and adjustment for multiple comparisons
☐	☒	A full description of the statistical parameters including central tendency (e.g. means) or other basic estimates (e.g. regression coefficient) AND variation (e.g. standard deviation) or associated estimates of uncertainty (e.g. confidence intervals)
☐	☒	For null hypothesis testing, the test statistic (e.g. *F*, *t*, *r*) with confidence intervals, effect sizes, degrees of freedom and *P* value noted<br>*Give P values as exact values whenever suitable.*
☒	☐	For Bayesian analysis, information on the choice of priors and Markov chain Monte Carlo settings
☒	☐	For hierarchical and complex designs, identification of the appropriate level for tests and full reporting of outcomes
☒	☐	Estimates of effect sizes (e.g. Cohen's *d*, Pearson's *r*), indicating how they were calculated

Our web collection on statistics for biologists contains articles on many of the points above.

Software and code

Policy information about availability of computer code

Data collection	No software was used for data collection.
Data analysis	FastQC v0.11.9; trimmomatic v0.39; Nanoplot v1.30; Porechop v0.2.3; NanoFilt v2.7.1; unicycler v0.4.8; snippy v4.6.0; nglmr v0.2.7; sniffles v1.0.12; Bandage v0.8.1; eggNOG-mapper v2; abricate v1; PHASTER (accessed in April 2020); islandviewer v4; circos v0.69, OmniLog data Analysis Software v1.7, JMP v16 software (SAS Statistics), mlst software v2.19.0

For manuscripts utilizing custom algorithms or software that are central to the research but not yet described in published literature, software must be made available to editors and reviewers. We strongly encourage code deposition in a community repository (e.g. GitHub). See the Nature Portfolio guidelines for submitting code & software for further information.

Data

Policy information about availability of data

All manuscripts must include a data availability statement. This statement should provide the following information, where applicable:

- Accession codes, unique identifiers, or web links for publicly available datasets
- A description of any restrictions on data availability
- For clinical datasets or third party data, please ensure that the statement adheres to our policy

Used Database(s) : NCBI BioProject database
Accession code : PRJNA776240

Link : https://www.ncbi.nlm.nih.gov/bioproject/PRJNA776240
Full Data Availability Statement : All sequencing data generated in this study has been deposited in the NCBI BioProject database accession PRJNA776240 [https://www.ncbi.nlm.nih.gov/bioproject/PRJNA776240]. Source data are provided with this paper.

Human research participants

Policy information about studies involving human research participants and Sex and Gender in Research.

Reporting on sex and gender	This work reports the case of a single 1 year old patient, of male sex determined by self-reporting and phenotypically confirmed upon clinical exam. The term "male toddler" is used in the manuscript to describe the patient. As this work is, as mentioned, a single-case report which did not imply any prospective "study design", sex and gender were not considered in this study. In the same way, data can not be represented disaggregated for sex and gender in this single-case study. We consider the experimental findings presented in this work irrelevant towards sex and gender since they are based on retrieved bacterial isolates, not on the human patient himself.
Population characteristics	One single patient.<br>Sex : Male<br>Age : 1 year old<br>Initial diagnosis of interest in this case : biliary atresia<br>Relevant treatment categories : post-transplantation long-term immunosuppressive drugs, broad-spectrum antibiotics
Recruitment	In the context of Salvage Therapy (under the article 37 of the Declaration of Helsinki).<br>The patient was not per se recruited to any prospective study ; the clinical case report part is a retrospective description of the patient's clinical story. Phage Therapy was proposed as a Salvage Therapy to the patient on the sole basis of his life-threatening systemic Pseudomonas aeruginosa infection, against which every option of standard-of-care antibiotic therapy had been previously tried, in vain. We do not acknowledge any bias in this process since phage therapy is an option that we will consider for any patient presenting a bacterial infection in such a state of standard-of-care therapeutic failure.
Ethics oversight	The Ethical Committee of the Saint-Luc University Hospital/UCLouvain, Brussels, Belgium<br>Written informed consent was obtained from both the patient's parents.

Note that full information on the approval of the study protocol must also be provided in the manuscript.

Field-specific reporting

Please select the one below that is the best fit for your research. If you are not sure, read the appropriate sections before making your selection.

☒ Life sciences ☐ Behavioural & social sciences ☐ Ecological, evolutionary & environmental sciences

For a reference copy of the document with all sections, see nature.com/documents/nr-reporting-summary-flat.pdf

Life sciences study design

All studies must disclose on these points even when the disclosure is negative.

Sample size	1 patient (single case retrospective report). As this work was not a prospective study but a single-case report, no sample size was predetermined.
Data exclusions	none
Replication	Galleria mellonella assay was first calibrated for optimal methodology of injection and follow-up on batches of five larvae each ; the assay was then performed on batches of ten larvae each, standardized for weight.<br>All other experiments were repeated at least three times.<br>All replications were considered successful by bringing satisfying results, none of which were excluded from final analysis.
Randomization	On the clinical part : randomization is a sorting process that is by definition not applicable to the single patient presented in this work.<br>On the experimental part : in the Galleria mellonella virulence assay, larvae were randomly allocated to any of the eight batches of ten larvae that were used for the assay.
Blinding	On the clinical part : clinical care teams including medical doctors, nurses and laboratory technicians (e.g. in clinical microbiology) were not blind to the single patient presented in this work.<br>On the experimental part : investigators were blind to allocation and outcome assessment in both the Galleria mellonella and the HeLa cells virulence assays. Investigators were not blind to allocation and outcome assessment in the OmniLog assays and in the Phage Immune Neutralization assay.

Reporting for specific materials, systems and methods

We require information from authors about some types of materials, experimental systems and methods used in many studies. Here, indicate whether each material, system or method listed is relevant to your study. If you are not sure if a list item applies to your research, read the appropriate section before selecting a response.

Materials & experimental systems

n/a	Involved in the study
☒	☐ Antibodies
☐	☒ Eukaryotic cell lines
☒	☐ Palaeontology and archaeology
☐	☒ Animals and other organisms
☒	☐ Clinical data
☒	☐ Dual use research of concern

Methods

n/a	Involved in the study
☒	☐ ChIP-seq
☒	☐ Flow cytometry
☒	☐ MRI-based neuroimaging

Eukaryotic cell lines

Policy information about cell lines and Sex and Gender in Research

Cell line source(s)	HeLa cells were obtained from Professor Johan Neyts, Laboratory of Virology and Chemotherapy, Rega Intitute KU Leuven, Leuven, Belgium
Authentication	None of the cell lines used were authenticated.
Mycoplasma contamination	Cell lines were not tested for Mycoplasma contamination.
Commonly misidentified lines (See ICLAC register)	No commonly contaminated or commonly misidentified cell line was used in the study.

Animals and other research organisms

Policy information about studies involving animals; ARRIVE guidelines recommended for reporting animal research, and Sex and Gender in Research

Laboratory animals	Species : Galleria mellonella. Strain : unspecified. Breeder origin : Netherlands. Stage : larva. Sex of the larvae : indifferent, uncharacterized. Age : ~3 weeks.
Wild animals	No wild animals were used in the study.
Reporting on sex	Sex of the larvae : indifferent, uncharacterized.
Field-collected samples	No field-collected samples were used in the study.
Ethics oversight	It was considered that the use of this inverterbrate larva model did not require specific ethical guidance.

Note that full information on the approval of the study protocol must also be provided in the manuscript.
